# Light- and Neutron-Optical Properties of Holographic Transmission Gratings from Polymer-Ionic Liquid Composites with Submicron Grating Spacing

**DOI:** 10.3390/polym11091459

**Published:** 2019-09-06

**Authors:** Peter Flauger, Mostafa A. Ellabban, Gašper Glavan, Jürgen Klepp, Christian Pruner, Tobias Jenke, Peter Geltenbort, Martin Fally

**Affiliations:** 1 Faculty of Physics, Physics of Functional Materials, University of Vienna, Boltzmanngasse 5, A-1090 Wien, Austria; 2 Physics Department, Faculty of Science, Tanta University, Tanta 31527, Egypt; 3 Physics Department, Faculty of Science, Taibah University, Medina 42353, Saudi-Arabien; 4 Faculty of Mathematics and Physics, University of Ljubljana, Jadranska 19, SI-1000 Ljubljana, Slovenia; 5 University of Salzburg, Department Chemistry and Physics of Materials, 5020 Salzburg, Austria; 6 Institut Laue-Langevin, 71 avenue des Martyrs CS 20156, CEDEX 9, 38042 Grenoble, France

**Keywords:** polymer composites, ionic liquids, diffraction gratings, neutron optics, thick gratings, holography

## Abstract

We investigate the applicability of polymer-ionic liquid composites as optical elements for light, as well as for slow neutrons. The gratings are recorded using two-beam mixing and are characterized experimentally based on their diffraction properties. We produced a set of samples differing in their thickness, ranging from 10 μm–100 μm. We demonstrate that it is possible to prepare transmission gratings with a lattice constant of Λ=480 nm, resulting in thick gratings for light, as well as neutrons. The presented samples show low optical losses in the Vis-UV spectrum and exhibit refractive index modulations of about $10^{-3}$ at λ=543 nm. However, further improvements have to be made to obtain efficient neutron optical components.

## 1. Introduction

Holographic materials and corresponding gratings with spacings in the micro- to sub-micrometer range are used as standard light diffractive optical elements [1,2], and they are also the subject of research concerning, e.g., holographic data storage [3] and waveguide couplers [4]. A variety of photosensitive materials has already been considered as the foundation for gratings applicable to light optics. Photorefractive crystals have the disadvantage of low refractive index modulations, but have excellent optical properties, as well as thermal and temporal stability in their favor. Photopolymers allow for much higher modulations, but usually suffer from their shrinkage during polymerization. Tomlinson et al. [5] introduced the idea of including non- or less reactive additives in the material. In this way, not only the density, but also the composition might be modulated, and the refractive index modulation increases. State-of-the-art materials are composites of polymers and liquid crystals [6,7,8] or nanoparticles [9,10,11,12]. The first are electrically switchable and highly anisotropic, while the latter offer extremely high refractive index modulations, as well as enhanced thermal stability [13] and reduced shrinkage compared to photopolymers. Further advances regarding shrinkage reduction were achieved by the inclusion of thiols into nanoparticle-polymer composites [10].

This work focuses on another composite material that makes use of ionic liquids as additives to a photopolymer [14,15,16]. In a previous work, the suitability of this polymer-ionic liquid composite as recording media for structures in the 5–10 μm range was examined [17]. The obtained results allow us to make use of the good resolution of the material in order to write thick holographic transmission gratings with a submicron grating spacing of Λ=480 nm. We prepared gratings for two different recording times and a series of thicknesses. The thicker samples (d≥85 μm) should not only be applicable for light, but also for (very) cold neutrons ($\lambda_{N}>1$ nm).

An article giving an overview of the advances in this field of research can be found in [18]. Lately, beam splitters and highly-efficient mirrors where produced from SiO${}_{2}$ nanoparticle composites [19,20,21]. Such optical elements could be used to build up an interferometer for cold neutrons, as discussed for instance in [22]. Concerning this application, the gratings are required to operate within the boundaries the Bragg regime (two-wave coupling regime), have high diffraction efficiencies (50% for beam splitters and 100% for mirrors), and exhibit low angular selectivity to reduce the effort put into the setup procedure of the device [23].

## 2. Materials and Methods

### 2.1. Materials’ and Sample Preparation

**Figure 1:**
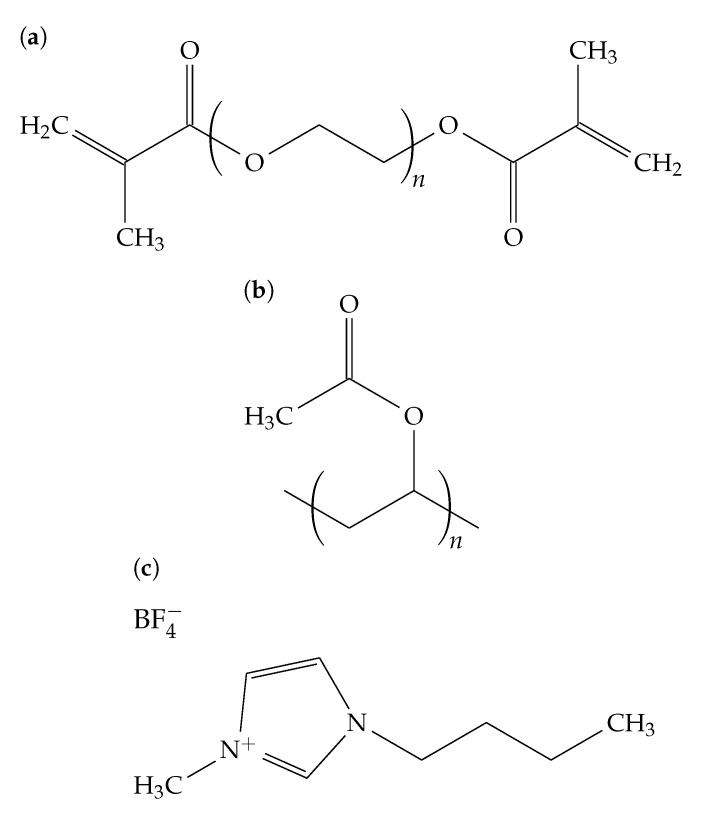
Structures of the components used: (**a**) PEGDMA, (**b**) PVAC, and (**c**) BMIMBF_4_.

The samples described in this work were made of a photopolymerizable polymer-ionic liquid composite suggested by Lin et al. in [14,15,16], inserted between two glass plates. The composite consisted of a polymer (poly-(ethylene glycol)-dimethacrylate (PEGDMA)), a polymer binder (polyvinyl acetate (PVAC)), and an ionic liquid (1-butyl-3-methylimidazolium tetrafluoroborate (BMIMBF${}_{4}$)) as an additive, as well as a UV-photoinitiator (Irgacure184 (Irg184)). The composition with respect to the weight ratio is given in Table 1. Their chemical structures are shown in Figure 1.

The produced samples differed in their thickness (10 μm, 15 μm, 20 μm, 50 μm, 85 μm, and 100 μm) and the exposure time ($t_{r}=7$ s or 9 s, respectively). Further details on the grating preparation were given in [24].

When the sample material was exposed to UV light, radical polymerization was initiated. Gratings could be holographically recorded by utilizing noncollinear two-beam interference. This led to a spatially-modulated illumination of the sample material. In the bright regions, the polymerization of monomers took place, and monomers from the dark regions diffused to the bright ones, while the photo-insensitive ionic liquids were expected to counter-diffuse [12]. This behavior lead to a density modulation of the constituents, which resulted in a refractive index modulation.

The gratings were recorded using a 355 nm solid-state laser and had a grating spacing of Λ=480 nm. The intensities of the two coherent beams were 8.4 mW/cm^2^ and 9 mW/cm^2^.

### 2.2. Sample Characterization

**Figure 2:**
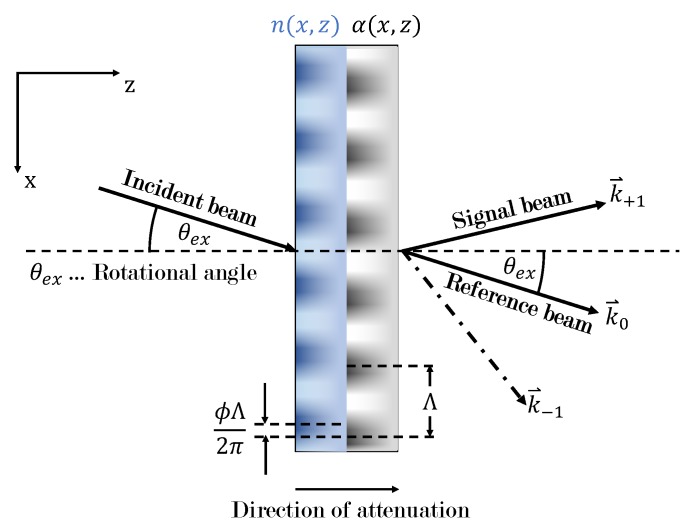
The gratings are assumed to consist of a phase and amplitude grating with mutually-shifted modulation. The modulation amplitudes $n_{1}\left(z\right)$ and $\alpha_{1}\left(z\right)$ are attenuated along the z-direction. In the experimental setup, the rotational angle of the sample corresponds to the external angle of incidence.

We modeled our samples as mixed gratings, i.e., consisting of a phase and amplitude grating, as illustrated in Figure 2. We assumed the modulation of the real-valued refractive index and the absorption coefficient to be sinusoidal along the x-direction and allowed for a phase shift $\phi =\phi_{n}-\phi_{\alpha }$ between phase and amplitude grating. The modulation amplitudes $n_{1}\left(z\right)=n_{1}\exp\left(-z/L\right)$ and $\alpha_{1}\left(z\right)=\alpha_{1}\exp\left(-z/L\right)$ were assumed to decay exponentially along the z-direction with 1/e-length *L*. Therefore, the complex-valued refractive index is given by:

(1) $$\begin{array}{cc} n_{c}\left(x,z\right) & =n\left(x,z\right)-\frac{i}{k_{0}}\alpha \left(x,z\right)\\ \; & =n_{0}+n_{1}\left(z\right)\cos\left(Gx-\phi_{n}\right)-i\left\{\frac{\alpha_{0}}{k_{0}}+\frac{\alpha_{1}\left(z\right)}{k_{0}}\cos\left(Gx-\phi_{\alpha }\right)\right\}, \end{array}$$

where $n_{0}$, $\alpha_{0}$, $k_{0}$, and *G* are the mean real part of the refractive index, the mean absorption coefficient, the wavenumber in free space, and the spatial frequency of the grating, respectively. The mean refractive index modulation along the z-direction is given by:

(2) $$\begin{array}{cc} \langle n_{1}\rangle & =\frac{n_{1}}{d}\int_{0}^{d}\exp\left\{-\frac{z}{L}\right\}dz\\ & =\frac{n_{1}}{d}L\left(1-\exp\left\{-\frac{d}{L}\right\}\right). \end{array}$$

**Figure 3:**
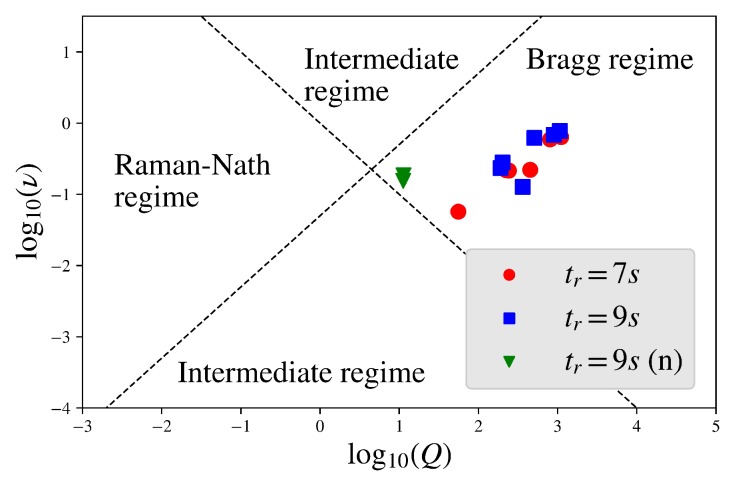
Classification of the samples into the diffraction regimes. Red markers resemble samples with a 7 s recording time and blue markers those with 9 s, both in the context of light diffraction. In contrast, green markers indicate neutron diffraction.

The characterization of our gratings was mainly done by carrying out light diffraction experiments. In these, the angular dependence of the diffraction efficiency $\eta_{j}\left(\theta \right)$ was measured. θ denotes the internal angle of incidence with respect to the surface normal of the sample and *j*th the order of the diffracted beam. Generally, we distinguished three diffraction regimes: the Raman–Nath regime, the Bragg regime, and an intermediate regime. The Bragg regime is characterized by the simultaneous occurrence of only two diffraction orders, while multiple orders appear at a given θ in the Raman–Nath regime, as if the Bragg condition were satisfied for all of them. We adopted the terminology of [25] and called a grating “thick” if its diffraction properties were governed by the Bragg regime, whereas “thin” meant the Raman–Nath regime. To chose a suitable theory for the analysis of the experimental data [26], we had to determine the diffraction regime first, as illustrated in Figure 3. The classification followed the guidelines of [25]: From a rigorous coupled wave analysis [27,28], it is known that certain ranges of values for a combination of the holographic parameters $\nu =\left(\pi \langle n_{1}\rangle d\right)/\left(\lambda \cos\left(\theta_{B}\right)\right)$ and $Q=\left(2\pi \lambda d\right)/\left(n_{0}\Lambda^{2}\cos\left(\theta_{B}\right)\right)$ decide the diffraction regimes: Qν<1andQ<20ν, as well as Qν>1andQ>20ν are indicators for the Raman–Nath regime and the Bragg regime, respectively. Since all our gratings will turned out to be thick, we applied a two-wave coupled wave diffraction theory allowing for a phase shift between the amplitude and phase grating [29,30] and a grating attenuation along the z-direction. We applied the beta-value method from [31] to the coupled wave theory introduced in [32]. This approach was described in [24]. Further details can be found in the Appendix A.

The diffraction efficiencies of the two beams (reference beam and signal beam corresponding to the zeroth and ±1st order, respectively) were calculated from the angular dependent amplitudes R(θ) and S(θ):

(3) $$\eta_{0}\left(\theta \right)=R\left(\theta \right)R^{*}\left(\theta \right)\;\mathrm{and}\;\eta_{\pm 1}=S\left(\pm \theta \right)S^{*}\left(\pm \theta \right)\frac{c_{S}\left(\pm \theta \right)}{c_{R}\left(\theta \right)}$$

where $c_{R}$ and $c_{S}$ are the projections of the corresponding beam’s unit vector on the z-axis. The model (see Equation (3)) can be fitted to the experimental data to estimate the grating’s thickness *d*, attenuation length *L*, Bragg angle $\theta_{B}$, refractive index modulation $n_{1}$, absorption modulation $\alpha_{1}$, and phase shift ϕ. In our case, the diffraction efficiency of a grating can be improved by increasing the thickness *d* or $\langle n_{1}\rangle$. However, increasing the thickness faces severe practical restrictions such as scattering and resulting grating attenuation. Therefore, a high value of $\langle n_{1}\rangle$ is desired.

The characterized gratings should also work as diffraction gratings for (very) cold neutrons. The refractive index for neutrons is given by:

(4) $$n_{N}\left(\vec{x}\right)=1-\frac{\lambda_{N}^{2}}{2\pi }b_{c}\rho \left(\vec{x}\right).$$

where $b_{c}$ denotes the coherent scattering length and $\rho (\vec{x})$ the number density (see for instance [33]). Consequently, the refractive index modulation for neutrons will increase with the square of $\lambda_{N}$, which makes long wavelengths desirable to work with.

## 3. Results

As already discussed in [24], the in-Bragg diffraction efficiency $\eta_{+1}\left(\theta_{B}\right)$ for light, acting as an indicator for the density modulation within the material, increases further for some time after exposure is terminated. It then passes a maximum and saturates. For this reason, the polymerization is expected to be diffusion-rate controlled, i.e., the diffusion-rate is higher than the polymerization-rate [14].

### 3.1. Light Diffraction Experiments

First, we wanted to examine the optical quality of the prepared samples. Absorbance measurements were performed using a CARY5G (Varian) spectrometer. Within the Vis-NIR range (between 400 nm and 800 nm), losses were smaller than 30% and mainly due to the glass–air interfaces. The spectral dependence of the absorbance was low; thus, the gratings were applicable for a wide range of wavelengths.

To characterize the gratings further, light diffraction experiments were carried out. Using a 543 nm He-Ne laser for readout, the angular dependence of the diffraction efficiency η(θ), the so-called rocking curve, was measured. If the mean absorption coefficient $\alpha_{0}$ was small, the diffraction efficiencies (3) were approximately:

(5) $$\eta_{0}\left(\theta \right)\approx \frac{I_{0}}{I_{0}+I_{\pm 1}}\;\mathrm{and}\;\eta_{\pm 1}\approx \frac{I_{\pm 1}}{I_{0}+I_{\pm 1}},$$

where $I_{j}$ denotes the intensity of the beam of order *m*. Since we expect $\alpha_{0}$ to be negligible, the efficiencies (3) are fitted to the data obtained by using the approximations (5).

We noticed that our experiments showed a strong inhomogeneity of the diffraction properties across the prepared grating area, i.e., the area illuminated during recording. The samples were scanned for the spot of maximum diffraction efficiency. The step width of the scanning procedure was around 0.2 mm, and the diameter of the beam used was about 2 mm. All data discussed and shown in this work were collected at the best spot.

**Figure 4:**
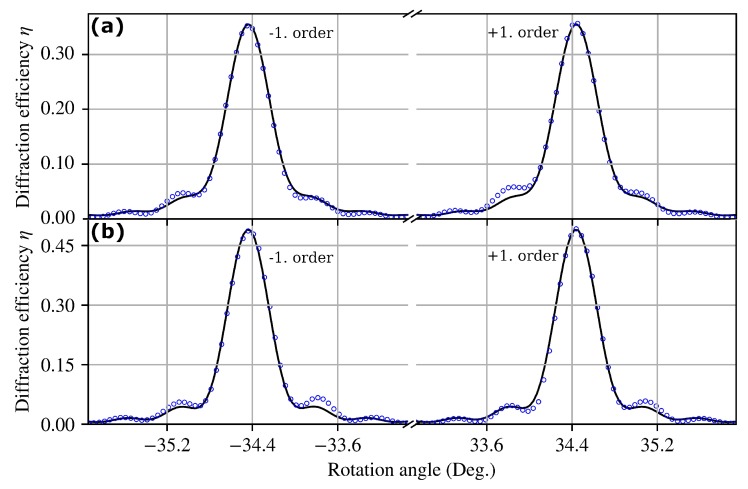
Angular dependence of the diffraction efficiency $\eta (\theta_{ex})$. $\theta_{ex}$ denotes the external angle of incidence of the beam with respect to the sample’s surface normal and corresponds to the rotation of the sample in the experimental setup. Symbols indicate measured data, while solid lines show the corresponding fits. The readout-laser wavelength was λ=543 nm. Both gratings had a lattice constant of Λ=480 nm and a thickness of d≈100 μm. (**a**) $t_{r}=7$ s, $n_{1}\left(0\right)=\left(2.72\pm 0.21\right)\times 10^{-3}$, d=101.5 μm ± 1.0 μm, L=40.92 μm ± 0.48 μm. (**b**) $t_{r}=9$ s, $n_{1}\left(0\right)=\left(2.77\pm 0.17\right)\times 10^{-3}$, d=98.10 μm ± 0.46 μm, L=53.21 μm ± 0.58 μm.

As indicated by Figure 3, where the optical parameters were compared, the diffraction properties were governed by the Bragg regime in the case of all gratings. Figure 4 shows rocking curves for two gratings with the same grating spacing and thickness, but with different exposure time $t_{r}$. Even though the theory presented in the Appendix A allows for mixed phase and amplitude gratings, a $\chi^{2}$ test or very poor uncertainty estimations of the fit parameters indicated that there was no considerable contribution of an amplitude grating in our samples or at least none that could be well distinguished from effects due to the grating attenuation. Table 2 contains the experimentally-measured values of the average refractive index modulation for all gratings.

### 3.2. Neutron Diffraction Experiments

**Figure 5:**
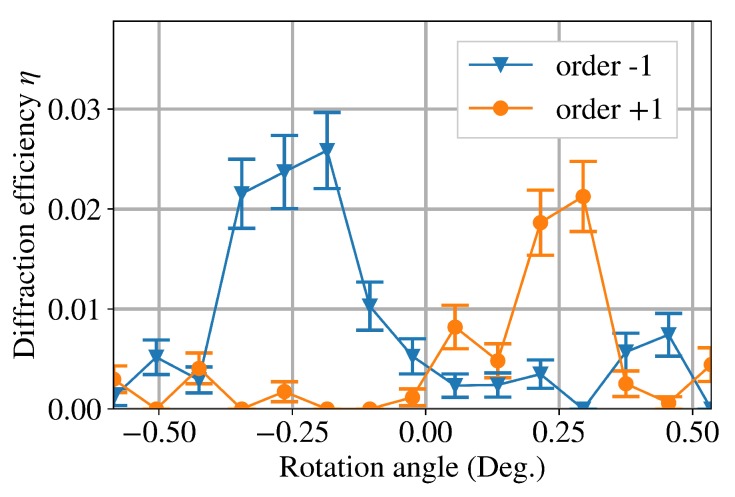
Measured angular dependence of the diffraction efficiency η for very cold neutrons with a central wavelength of around 3 nm [34]. Light diffraction properties of the same grating ($t_{r}=9$ s, d≈100 μm) are shown in Figure 4b. Note that, for neutrons, $\theta \approx \theta_{ex}$.

The neutron optical measurements were performed at the Institut Laue-Langevin, beamline PF2/VCN, Grenoble. The neutrons had a broad wavelength distribution between 1 nm and 9 nm with a central wavelength of roughly 3 nm. The experiments were carried out for the samples with d≈100 μm and d≈85 μm from the $t_{r}=7$ s and $t_{r}=9$ s sets. While no diffraction signal was found for the $t_{r}=7$ s samples, small diffraction efficiencies (<3% for d≈100 μm and <4% for d≈85 μm) were found for the $t_{r}=9$ s samples. Figure 5 shows the rocking curves for $t_{r}=9$ s and d≈100 μm.

## 4. Discussion

Light optical diffraction experiments showed that the investigated gratings had a mean refractive index modulation $\langle n_{1}\rangle$ of around $1.3\times 10^{-3}$ ($t_{r}=7$ s) and $1.6\times 10^{-3}$ ($t_{r}=9$ s), respectively. This was one order of magnitude less than for gratings recorded in nanoparticle-polymer composites, but much higher than the values in the order of $10^{-5}$ achieved with photorefractive crystals [12,35]. By comparing $\langle n_{1}\rangle$ as given by Equation (2) instead of $n_{1}$, we favored samples with smaller *d* to some extent. Furthermore, one should remember that the practical range of the thickness *d* is limited by holographic scattering.

The achieved diffraction efficiencies for light (λ=543 nm) from 30% up to almost 50% for the thicker samples (d=85 μm and 100 μm) made for a satisfactory result, showing that it was indeed possible to write efficient gratings with low losses and submicron grating spacing in the polymer-ionic liquid composite used. It is also noteworthy that the $t_{r}=9$ s gratings showed in general higher efficiencies than their shorter illuminated counterparts.

Still, we noticed a troublesome inhomogeneity of the grating quality across the recording area, similar to as shown in an earlier work (Figure 10 of [17]). In order to make use of the materials investigated in this work for reliable optical elements, this inhomogeneity has to be reduced. However, its source is not known, but could arise at least in part from an inhomogeneous segregation of components. Investigations on the observed inhomogeneity are on the way. One could try to replace the UV photoinitiator with one in the visible range to make efficient use of the higher powers available with lasers of visible wavelengths. It remains to be tested how different combinations of power and recording time affect the resulting modulation in a diffusion-rate-controlled polymerization process [17]. An additional problem arises with thicker samples in the form of air or other inclusions between the sample material and its glass coating due to shrinkage during polymerization.

## 5. Conclusions

The achieved grating spacing was an encouraging result since, in principle, it allowed for neutron optical elements with diffraction taking place in the Bragg regime, which is desirable for two-port devices. However, the obtained diffraction efficiencies were not satisfactory for practical usage at the current stage of development and cannot compete with gratings recorded in nanoparticle-polymer composite [12,18,20]. This might be attributed in part to the strong inhomogeneity or the possibility that the density modulation was too small and that the refractive index modulation observed for light was mainly due to the differences in polarizability.

## Figures and Tables

**Table 1:** Composition of the sample material.

Material	Percentage (wt%)	Description
poly-(ethylene glycol)-dimethacrylate (PEGDMA)	73.1	Polymer
polyvinyl acetate (PVAC)	7.3	Polymer binder
1-butyl-3-methylimidazolium tetrafluoroborate (BMIMBF${}_{4}$)	18.2	Ionic liquid
Irgacure184 (Irg184)	1.4	UV photoinitiator

**Table 2:** Average light refractive index modulation $\langle n_{1}\left(z\right)\rangle$.

thickness *d*	$\langle n_{1}\rangle \left(t_{r}=7\right)$	$\langle n_{1}\rangle \left(t_{r}=9\right)$
10 μm	$\left(1.512\pm 0.088\right)\times 10^{-3}$	$\left(2.142\pm 0.044\right)\times 10^{-3}$
15 μm	$\left(1.536\pm 0.071\right)\times 10^{-3}$	$\left(2.411\pm 0.024\right)\times 10^{-3}$
20 μm	$\left(1.692\pm 0.025\right)\times 10^{-3}$	$\left(6.319\pm 0.085\right)\times 10^{-4}$
50 μm	$\left(8.53\pm 0.14\right)\times 10^{-4}$	$\left(2.125\pm 0.040\right)\times 10^{-3}$
85 μm	$\left(1.277\pm 0.022\right)\times 10^{-3}$	$\left(1.327\pm 0.031\right)\times 10^{-3}$
100 μm	$\left(9.98\pm 0.18\right)\times 10^{-4}$	$\left(1.263\pm 0.013\right)\times 10^{-3}$

## Abbreviations

The following abbreviations are used in this manuscript:

BMIMBF${}_{4}$	1-butyl-3-methylimidazolium tetrafluoroborate
PEGDMA	poly-(ethylene glycol)-dimethacrylate
PVAC	polyvinyl acetate

## Appendix A

Here, we present a two-wave coupled wave diffraction theory allowing for a phase shift between the amplitude and phase grating [29,30] and a grating attenuation along the z-direction. Therefore, the beta-value method [31], which sets the wavenumber of both occurring beams to $\beta =n_{0}k_{0}$, is applied to the coupled wave theory introduced by Kogelnik [24,32]. In order to do so, we introduce a mismatch between the z-components of the k-vectors of the reference and signal beam within the grating region:

(A1) $$\begin{array}{cc} \Delta k & =k_{R,z}-k_{S,z}\\ \; & =\beta (c_{R}-c_{S}) \end{array}$$

(A2) $$\begin{array}{cc} c_{R} & =\cos\left(\theta \right) \end{array}$$

(A3) $$\begin{array}{cc} c_{S} & =\sqrt{1-{\left(\left|\sin\left(\theta \right)\right|-\frac{G}{\beta }\right)}^{2}}. \end{array}$$

As the wavenumbers of both beams within the grating were fixed to β, energy conservation was assured. One can now go on to solve the Helmholtz equation with a superposition of reference and the signal wave as the ansatz:

(A4) $$\psi =R\left(z\right)\exp\left(-i{\vec{k}}_{R}\vec{x}\right)+S\left(z\right)\exp\left(-i{\vec{k}}_{S}\vec{x}\right),$$

with R(z) and S(z) being the amplitudes we want to calculate at the interface at z=d. After using as usual slowly-varying amplitude approximation, we end up with a set of two first order coupled differential equations, namely:

(A5) $$\begin{array}{cc} \alpha_{0}R\left(z\right)+c_{R}R^{\prime }\left(z\right) & =-i\kappa^{-}\left(z\right)S\left(z\right)\exp\left\{i\left(\phi_{n}+z\Delta k\right)\right\} \end{array}$$

(A6) $$\begin{array}{cc} \alpha_{0}S\left(z\right)+c_{S}S^{\prime }\left(z\right) & =-i\kappa^{+}\left(z\right)R\left(z\right)\exp\left\{-i\left(\phi_{n}+z\Delta k\right)\right\} \end{array}$$

that can be decoupled and solved using the boundary conditions for a transmission grating: S(0)=0andR(0)=1. The amplitudes read:

(A7) $$\begin{array}{cc} R(z)= & \frac{\pi }{4a\sin\left(\pi \omega^{*}\right)}\exp\left\{-\frac{1}{2}\left(a+d_{R}\right)z+i\phi_{n}\right\}\\ & \times [J_{\omega^{*}}\left(\nu e^{-az}\right)\left\{2bJ_{1+\omega^{*}}\left(\nu \right)-4a\omega^{*}J_{\omega^{*}}\left(\nu \right)\right\}\\ \; & -2bJ_{1-\omega^{*}}\left(\nu \right)J_{\omega^{*}}\left(\nu e^{-az}\right)] \end{array}$$

(A8) $$\begin{array}{cc} S(z)= & -\frac{i\pi \kappa^{+}\left(0\right)}{a\sin\left(\pi \omega \right)}\exp\left\{-\frac{1}{2}\left(a+d_{S}\right)z-i\phi_{n}\right\}\\ \; & \times \left[J_{-\omega }\left(\nu e^{-az}\right)J_{\omega }\left(\nu \right)-J_{-\omega }\left(\nu \right)J_{\omega }\left(\nu e^{-az}\right)\right]. \end{array}$$

Here, we introduced the abbreviations:

(A9) $$\begin{array}{c} a=\frac{1}{L} \end{array}$$

(A10) $$\begin{array}{c} \kappa^{\pm }\left(z\right)=\frac{\pi }{\lambda }n_{1}\left(z\right)-i\frac{\alpha_{1}\left(z\right)}{2}\exp\left(\pm i\phi \right) \end{array}$$

(A11) $$\begin{array}{c} b^{2}=\frac{\kappa^{+}\left(0\right)\kappa^{-}\left(0\right)}{c_{R}c_{S}} \end{array}$$

(A12) $$\begin{array}{c} d_{R}=\alpha_{0}\left(\frac{1}{c_{S}}+\frac{1}{c_{R}}\right)-i\Delta k\;\;d_{S}=\alpha_{0}\left(\frac{1}{c_{S}}+\frac{1}{c_{R}}\right)+i\Delta k \end{array}$$

(A13) $$\begin{array}{cc} f_{R}=\frac{\alpha_{0}}{c_{R}}\left(\frac{\alpha_{0}}{c_{S}}+a-i\Delta k\right) & \;\;f_{S}=\frac{\alpha_{0}}{c_{S}}\left(\frac{\alpha_{0}}{c_{R}}+a+i\Delta k\right) \end{array}$$

$J_{\omega }\left(\nu \right)$ denotes the Bessel function and:

(A14) $$\begin{array}{cc} & \omega =\frac{1}{2a}\left[\alpha_{0}\left(\frac{1}{c_{R}}-\frac{1}{c_{S}}\right)+a+i\Delta k\right] \end{array}$$

(A15) $$\begin{array}{cc} & \nu =\frac{b}{a}. \end{array}$$

The diffraction efficiencies can be calculated from the amplitudes in Equation (A8).

(A16) $$\eta_{0}\left(\theta \right)=R\left(\theta \right)R^{*}\left(\theta \right)\;\mathrm{and}\;\eta_{\pm 1}=S\left(\pm \theta \right)S^{*}\left(\pm \theta \right)\frac{c_{S}\left(\pm \theta \right)}{c_{R}\left(\theta \right)}$$

Equation (3) can be fitted to the experimental data to characterize the gratings.
